# Supplementary material for: Patterns of Intron Gain and Loss in Fungi
Source: PLoS Biol. 2004 Nov 30;2(12):e422. doi: 10.1371/journal.pbio.0020422 (PMC532390; doi:10.1371/journal.pbio.0020422)
Supplement: Table S1 — Also available at http://genes.mit.edu/NielsenEtAl/. (4.3 MB ZIP). [file pbio.0020422.st001.zip › NielsenEtAl/html/1335.html]

AN0127.1.NCU03441.1.MG03139.1.FG04297.1


```
 CLUSTAL W (1.82) Multiple Sequence Alignments - Introns Inserted


Sequence 1: MG03139.1	469 aa
Sequence 2: FG04297.1	459 aa
Sequence 3: NCU03441.1	461 aa
Sequence 4: AN0127.1	444 aa
Alignment Length: 519 aa
Number Identitical Residues: 213 aa
Alignment Score (without introns) 10130


MG03139.1 	MATATLNFITFNQDHGCLAVG1TSRGFRIYHTEPFSKIFSSEDGNVSIIEMLFSTSLVAL
NCU03441.1	MTGGTLNFITFNQDYSCLAVG~TSKGFRFYHTDPFSKIFSSDEGNVSIIEMLFSTSLVAL
FG04297.1 	MTSPALNFITFNQDHSCLAVG1TSKGFRIYHTDPFSRIFSSDDGNIAIIEMLFSTSLVAL
AN0127.1  	---MTMNFVTFNQDYSYLAVA1TSKGFRIFTTDPFAKSYETKDGNIAIIEMLFSTSLVAL
          	    ::**:*****:. ***. **:***:: *:**:: :.:.:**::*************

MG03139.1 	ILSPRHLIIQN-------------------------TK~RGSVICELTFPSAVLAVRLNR
NCU03441.1	ILTPRQLEIQNTKVDQPSQRHCLASELRLMRRPILPTQ~RASVICELTFPSAVLAVRLNR
FG04297.1 	ILSPRHLIIQN-------------------------TK0RASTICELTFPSAVLAVRLNR
AN0127.1  	ILSPRRLQITN-------------------------TK0RQSTICELTFPTTVLAVKLNR
          	**:**:* * *                         *: * *.*******::****:***

MG03139.1 	KRLAVVLEDEIYLYDIANMSLLFTIATSPNPS---------------AICALSPSSENCF
NCU03441.1	KRLAVVLECQIYLYDVSNMNLVQTIDTSPNPN---------------AICALSPSSDSCY
FG04297.1 	KRLAVVLEEEIYLYDISNMSLLHTIATSPNPS---------------AICALSPSSENCF
AN0127.1  	KRLVIVLEDQIYLYDIQTMKLLYTIETSPNPNGKVSVQLNEPSLTPTALCALSPSSENCY
          	***.:*** :*****: .*.*: ** *****... : . ...: :.:*:*******:.*:

MG03139.1 	LAYPLPKPREDKDDKRPSHAPPLPTYIPPTSGDVLIFDAITLKAVNVIEAHRSPLSCIAI
NCU03441.1	LVYPRPNPREDVGAKAPAHLPPPSQYVPPKRGDVLVYDALNLKTVNVVEAHKSPLCAIAL
FG04297.1 	IAYPLPKPREDPDANRPAHAPPQSTFVAPTSGEVLIFDTLSLKAVNVIEAHRSPLCCICL
AN0127.1  	LAYPLPQKAAPSSFNPPAHAPPGNTHVSPTSGEVLIFDTLKLEAINVIEAHRSPLACITL
          	:.** *:     . : *:* **   .:.*. *:**::*::.*:::**:***:***..* :

MG03139.1 	NSEGTLLATASETGTIIRVFTVPKGQKLYQFRRGTYPSTIYSMSFNLSSTLLCVSSTSDT
NCU03441.1	NHDGSMLATASETGTIIRVFSLPQGQKLFQFRRGTVPTSIYSMSFNLSSTLLCVSSTSDT
FG04297.1 	NNEGTLLATASETGTIIRVFSVPKGKKLYQFRRGTYPSTIYSMSFNLSSTLLCVSSTSDT
AN0127.1  	NSDGTLIATASDKGTIIRVFSVPDGHKLYQFRRGSIPSRIFSMSFNTTSTLLCVSSSTET
          	* :*:::****:.*******::*.*:**:*****: *: *:***** :********:::*

MG03139.1 	VHIFRLGGPNNGASGAAGAGSAGEVLAASPGQDITGSPRADRWSRSRSYDSGNESPGSGS
NCU03441.1	VHIFRLLNTQKNANALPGGG-------------------PAQGTRSRSFDVNDPSPHKGE
FG04297.1 	IHIFRLGAPP-GNTTPAGAP------IESP-----ASQRQDRWSRARSYDD-SESPG-AS
AN0127.1  	IHLFKLSQPSQLQETSSANT--------------------SSTGRRRSLSSLSQSPE-RE
          	:*:*:*  . .     ..                          * ** .  . **   .

MG03139.1 	EANDIAGSPSPRDRPTAANR-----RQSGSFSNILRRSSQIMGRSVAGVVGSYLPQTVTE
NCU03441.1	VPSAGNGN----NGSGSHKR-----EPSGSFGNMLRRSSQMMVRGVAGVASSYLPQSVAE
FG04297.1 	AADSPKNEPAELNGPGAGNNQGGHTRGSGSFSSMLRRSSQIMGRGVAGVMGSYLPQSVTE
AN0127.1  	ATEEDNGS------SDLASR-----KHNGTLMGMLRRTSQNVGGAFAAKVGGYLPKGVSE
          	 ..   ..      .   ..     . .*:: .:***:** :  ..*.  ..***: *:*

MG03139.1 	MWEPARDFAFIKIPKSSAARQHNNPGATPSLPIA-GEPLRSVVAMSSSSPQVMVVTSDGK
NCU03441.1	MWEPERDFAFIKIPKSSNN-------RAGGLSAT---PLRSVVAMSSSSPQVMVVTSDGG
FG04297.1 	MWEPLRDFAYIKIPKSAAA-----SGASRTLRDAPGGPLRSVVAMSSSSPQVMVVTSDGG
AN0127.1  	MWEPARDFAWIKIPKPNQG-------QGPNANTG---PLRSVVAMSSNTPQVMVVTSDGN
          	**** ****:*****.                     **********.:********** 

MG03139.1 	FYVYNINMETGGEGYLVRQY-~---S2ILENDDKHDSSSTYES
NCU03441.1	FYVYNIDMEKGGEGYLVKQF-~---S2VLDGDDKLTNSD-YGA
FG04297.1 	FYVYNIDMEHGGEGYLVKQFS2VNHS~VLEGDDKSDASG-YGS
AN0127.1  	FYVFSIDLSKGGEGTLTKQY-~---S2VLESNDRLGYSVADY-
          	***:.*::. **** *.:*:     * :*:.:*:   * :
```
